# Supplementary material for: Incidence and Predictors of Mortality among Community-Dwelling Older Adults in Malaysia: A 5 Years Longitudinal Study
Source: Int J Environ Res Public Health. 2022 Jul 22;19(15):8943. doi: 10.3390/ijerph19158943 (PMC9331297; doi:10.3390/ijerph19158943)
Supplement: Supplementary file 1 [file ijerph-19-08943-s001.zip › ijerph-1753619-supplementary.pdf]

International Journal of
Environmental Research and Public Health

MDPI

Supplementary Materials

Table S1. Nutrient intakes of the respondents according to gender.

Nutrients at Baseline	Alive	Died	RNI 2017	*p*-Value
**Men**	(*n* = 909)	(*n* = 205)		
Energy (kcal)	1802 ± 465	1714 ± 461	1550–1780	0.014 *
Carbohydrates (g/day)	250.98 ± 77.88	236.01 ± 75.61		0.147
Protein (g/day)	74.69 ± 20.68	72.37 ± 20.77	50–58	0.013 *
Fat (g/day)	54.96 ± 18.73	54.48 ± 25.99		0.762
Total fibre (g/day)	3.92 ± 2.38	3.39 ± 2.09	20–30	0.003 *
Vitamin A (RE/day)	1228.06 ± 810.36	1191.66 ± 776.74	600	0.558
Vitamin C (mg/day)	117.31 ± 79.96	102.37 ± 80.08	70	0.016 *
Vitamin D (mg/day)	0.38 ± 3.00	0.21 ± 0.69	0.015–0.02	0.445
Vitamin E (mg/day)	6.82 ± 25.79	11.30 ± 67.77	7.5–10.0	0.120
Vitamin K (mg/day)	17.20 ± 58.70	11.82 ± 46.28	55–65	0.219
Thiamin (mg/day)	1.65 ± 3.80	1.20 ± 2.71	1.1–1.2	0.048 *
Riboflavin (mg/day)	1.26 ± 0.45	1.18 ± 0.49	1.1–1.3	0.027 *
Niacin (mg/day)	10.76 ± 3.87	10.29 ± 3.70	14–16	0.108
Cobalamine (µg/day)	3.93 ± 3.50	4.19 ± 3.38	4.0	0.331
Pyridoxine (mg/day)	0.73 ± 0.35	0.73 ± 0.40	1.5–1.7	0.923
Folate (µg/day)	106.80 ± 68.30	94.53 ± 60.29	400	0.018 *
Calcium (mg/day)	532.80 ± 232.97	504.77 ± 228.71	1000	0.119
Iron (mg/day)	14.07 ± 5.00	13.45 ± 5.57	11–14	0.117
Selenium (µg/day)	24.72 ± 18.64	20.34 ± 16.73	23	0.002 *
Zinc (mg/day)	3.84 ± 1.91	3.85 ± 2.39	4.4	0.950
Copper (mg/day)	0.59 ± 0.32	0.56 ± 0.34	0.9	0.235
Magnesium (mg/day)	133.56 ± 59.32	128.61 ± 57.49	420	0.278
**Women**				
Energy (kcal)	1529 ± 423	1402 ± 447	1550–1780	0.001 *
Carbohydrates (g/day)	201.32 ± 63.56	186.71 ± 61.90	50–58	0.013 *
Protein (g/day)	67.06 ± 21.37	61.60 ± 20.84	20–30	0.006 *
Fat (g/day)	50.60 ± 19.01	45.92 ± 21.51		0.009 *
Total fibre (g/day)	4.02 ± 2.50	2.92 ± 1.64		<0.001 *
Vitamin A (RE/day)	1169.47 ± 755.97	1118.15 ± 1017.50	600	0.482
Vitamin C (mg/day)	117.22 ± 81.91	91.78 ± 60.60	70	0.001 *
Vitamin D (mg/day)	0.31 ± 0.93	0.28 ± 1.38	0.015–0.02	0.801
Vitamin E (mg/day)	14.59 ± 69.71	4.45 ± 3.16	7.5–10.0	<0.001 *
Vitamin K (mg/day)	19.26 ± 73.38	10.87 ± 25.36	55–65	0.008 *
Thiamin (mg/day)	1.45 ± 3.25	1.60 ± 3.75	1.1–1.2	0.623
Riboflavin (mg/day)	1.20 ± 0.50	1.10 ± 0.50	1.1–1.3	0.037 *
Niacin (mg/day)	9.97 ± 3.86	9.11 ± 3.49	14–16	0.016 *
Cobalamine (µg/day)	3.82 ± 3.61	3.42 ± 2.83	4.0	0.219
Pyridoxine (mg/day)	0.67 ± 0.34	0.58 ± 0.34	1.5–1.7	0.004 *
Folate (µg/day)	104.46 ± 76.28	92.05 ± 74.32	400	0.078
Calcium (mg/day)	501.79 ± 238.42	474.74 ± 285.89	1000	0.231
Iron (mg/day)	12.86 ± 5.28	11.76 ± 5.60	11–14	0.025 *
Selenium (µg/day)	23.81 ± 17.59	20.41 ± 15.14	23	0.034 *
Zinc (mg/day)	3.40 ± 1.80	3.03 ± 2.10	4.4	0.029 *
Copper (mg/day)			0.9	0.002 *
Magnesium (mg/day)			420	0.001 *

* Significant at $p < 0.002$ using Independent *t*-test.
